# Zinc and ageing: third Zincage conference

**DOI:** 10.1186/1742-4933-4-5

**Published:** 2007-09-20

**Authors:** Eugenio Mocchegiani

**Affiliations:** 1 Immunolgy Ctr. (Section Nutrition, Immunity and Ageing) Res. Dept. INRCA, Ancona, Italy

## Abstract

The importance of Zn for optimal functioning of the immune system and antioxidant stress response is well documented. Zn homeostasis influences development and function of immune cells, activity of stress-related and antioxidant proteins [metallothioneins (MT), chaperones, ApoJ, Poly(ADP-Ribose) polymerase-1 (PARP-1) and Methionione Sulfoxide Reductase (Msr), Superoxide Dismutase (SOD)], and helps to maintain genomic integrity and stability. During ageing, the intake of Zn decreases due to inadequate diet and/or intestinal malabsorption, contributing to frailty, general disability and increased incidence of age-related degenerative diseases (cancer, infections and atherosclerosis). Although many factors contributing to Zn deficiency have been identified, the biochemical markers of Zn deficiency as well as the possibility to achieve relevant health benefits through Zn supplementation in the elderly are still a matter for evaluation. Taking into account that Zn homeostasis is regulated by proteins and enzymes for which polymorphisms have been previously found to be associated with successful/unsuccessful ageing, genetic screening might be of added value in evaluating the individual response to Zn supplementation. Biochemical, immunological, dietary and genetic studies aimed at understanding the impact of Zn in healthy ageing, the effect of Zn supplementation in the elderly and finally formulating a rationale for the promotion of correct Zn supplementation were discussed at the international Zincage conference held in Ancona in January 2007.

## Introduction

**Zincage **[1] is a specific targeted research project (STREP) funded by the European Union in the 6th Framework Program (FP6). It includes epidemiological studies on the influence of diet and lifestyle on healthy ageing, aimed at preventing adult degenerative disease, particularly focusing on cardiovascular diseases and also addressing malnutrition of the elderly. The conference held in Ancona, January 2007, was focussed particularly on the effects of Zn supplementation in the elderly and on the possible influences of dietary, biochemical and genetic factors on the individual response. Zn deficiency, cell-mediated immune dysfunction and increased oxidative stress are common in elderly subjects and it is quite clear that dietary habits including Zn consumption have a great impact on these factors. Zn supplementation in the elderly can improve the immune response and reduce oxidative stress markers, thereby contributing to a reduced incidence of infections. However, individual differences in the response to Zn can lead to contradictory results even with supplementation trials performed in elderly people of the same age-groups. One of the reasons for these individual differences is the different genetic background of the subjects enrolled in the study. In fact, some proteins with documented polymorphic sites are involved in regulating Zn homeostasis. One important class of such proteins are the metallothioneins (MT), which bind Zn with high affinity but, at the same time, release free Zn ions in response to oxidative/nitrosative stress and thereby modulate the expression of Zn-dependent genes and activate antioxidant enzymes. Differences in Zn status have also been observed in individuals carrying different alleles for polymorphisms of pro-inflammatory cytokines (i.e. IL-6 and TNF-alpha). In addition, the individual response can be modulated by dietary habits because Zn absorption and availability is dependent on the intake of other nutrients and trace elements.

These aspects have been poorly studied because the intracellular mechanisms involved in the regulation of Zn homeostasis are not well understood in the context of aging. Moreover, even if it is known that Zn can modulate antioxidant responses, very little, if any, focused research aimed at identifying the targets of the intracellular response in ageing has been undertaken. What is known is that during ageing, the intake of Zn decreases, thus contributing to frailty, general disability and increased incidence of age-related degenerative diseases (cancer, infections and atherosclerosis). This situation may be worsened or ameliorated in different European countries, due to the large differences in extrinsic (dietary habits and socio-economic conditions) and intrinsic factors (genetic background) affecting Zn homeostasis in southern and northern European countries. One of the aims of the Zincage project was to investigate how these factors affect the response to Zn, and thus to contribute to better evaluation of the need for this supplement in elderly populations.

## Zn, genetic background and longevity

Recent advances in understanding molecular and biological processes, evolutionary biology, and epidemiological data strongly suggest that human longevity is the product of genetic, environmental and stochastic interactions. What has been identified as an unfavourable genetic background for the risk of age-related disease and for longevity in some geographic areas is often not confirmed by studies performed in different regions. This suggests that environmental factors strongly interact with the individual genetic background. Different studies on European dietary habits confirm that the intake of micronutrients, including Zn, is different among different European countries. Moreover, the products of some genes with documented polymorphic sites are involved in regulating Zn homeostasis and inflammatory response, thereby affecting the propensity to develop Zn deficiency at advanced age. This means that in order to perform appropriate Zn supplementation in the elderly, it is necessary to take into account the Zn status, dietary habits and the individual genetic background of each person [2]. This last point may be crucial also in order to identify subjects that are at higher risk to develop Zn deficiency in aging. Here, then, there is potential for a preventive intervention with Zn supplementation as suggested by **Eugenio Mocchegiani (INRCA-Italian National Research Centres on Aging, Italy)**, who reported variability in the response to Zn supplementation according to IL-6-174 and MT1A +647 polymorphisms as well as to country of origin. Zn supplementation (48 ± 2 days) at a dose of 10 mg/day as Zn aspartate (Unizink 50, Kohler Pharma GmbH, Germany) induced a general increase of Zn levels in the whole supplemented sample (n = 148 elderly), but subjects harbouring different genotypes showed different responses both in the increases of plasma Zn and the intracellular Zn status. This finding becomes of paramount importance when we consider the well known role of Zn homeostasis in regulating inflammatory responses [3]. In fact, according to the antagonistic pleiotropy theory of ageing, natural selection has favoured genes conferring short-term benefits to the organism at the cost of deterioration in later life. Inflamm-aging, the low-grade, chronic, systemic inflammatory state that characterizes the aging process, is just an example of the antagonistic theory as explained by **Claudio Franceschi (University of Bologna, Italy)**. Franceschi reviewed recent advances in understanding the unusual genetics of human longevity, pointing out that a major contribution derives from pro-inflammatory and anti-inflammatory genes generally associated with age-related diseases [4]. Accordingly, **Calogero Caruso (University of Palermo, Italy) **reviewed the reports indicating an association between the risk of Alzheimer Disease (AD) and polymorphisms of genes encoding inflammatory mediators, such as IL-1β, IL-6, IL-10 and TNF-α [5], as well as the possible involvement of Zn. In this respect, the presence of Zn dyshomeostasis in the AD brain could be related to the dysregulation of inflammatory mediators in AD patients. However, clear evidence for a possible interplay between dietary Zn and risk of AD is still missing.

## Zn and brain

In neurodegenerative diseases, especially AD, a growing literature suggests pathogenic links between Zn and pathways involved in neurodegeneration. While Zn supplementation has beneficial antinflammatory effects, it can also play a pivotal role in the polymerization of Aβ peptide, and plaque formation. Therefore, when supplementating the elderly with Zn, one should always keep in mind that excessive Zn might have very serious deleterious effects. To address the neurotoxic role of Zn in neurological diseases, **Stefano Sensi (University G. D'Annunzio, Italy) **showed how an excessive increase in intracellular free Zn^++^, either by influx through glutamate ionotropic receptor-associated channels or the mobilization of the cation from MTs and mitochondria, can act as a potent catalytic factor in exacerbating neuronal death in response to excitotoxic levels of glutamate [6]. The excitotoxic cascade by which Zn exerts its neurotoxicity includes mitochondrial and extra-mitochondrial production of reactive oxygen species and disruption of metabolic enzymatic activity, ultimately leading to neuronal necrosis and/or apoptosis. Therefore, not only deficit but also excess of zinc is a condition that may be detrimental, especially for neuronal cells, which are particularly sensitive to oxidative stress. However, the precise role of zinc in AD and other neurodegenerative diseases is difficult to define because these diseases are progressive, apparently irreversible and fatal, so that it is very difficult to discriminate causal or contributing factors from compensatory defensive mechanisms. **Paolo Zatta and Denise Drago (University of Padova, Italy) **showed the aggregation kinetic of amyloid-beta after exposure to trace elements (Al, Zn, Fe and Cu) [7]. The most dramatic effects were observed with Al^3+^, and to a lesser extent with Zn, which in turn induced the formation of unstructured filaments which were less toxic than the ones induced by Al^3+^. Ageing is also associated with a progressive decrease of mitochondrial metabolic competence with the appearance of a kind of mosaicism characterized by increased mitochondria with metabolic impairments or complete metabolic deficiency [8]. **Carlo Bertoni (INRCA-Italian National Research Centres on Aging, Italy) **illustrated this phenomenon in association with the age-related changes in the synaptic structural dynamics with a particular focus on the mitochondrial targets of Zn. These targets include complexes of the mitochondrial electron transport chain, components of the tricarboxylic acid cycle, and enzymes of glycolysis. An inhibitory effect of Zn on these targets is most likely, but the relevance of this phenomenon in ageing remains to be investigated appropriately.

## Zn and oxidative stress

A number of Zn-dependent stress-related proteins (PARP-1, ApoJ, MT, NO, chaperones) and the activity of Zn-dependent (SOD) and other (GPX and catalase) antioxidant enzymes in the elderly, as well as the role of the proteasomal system in the degradation of proteins whose abnormal accumulation is harmful in ageing, as well as the role of methionine sulfoxide reductase (Msr) in repairing oxidised proteins were all investigated during the Zincage Project. **Alexander Buerkle and Andrea Kunzmann (University of Konstanz, Germany) **showed that cellular poly(ADP-ribosyl)ation is positively correlated with Zn status in human peripheral blood mononuclear cells (PBMC), and confirmed that an age-related decrease of PARP-1 occurs with "in vitro" (T Cell Clone model) and "in vivo" ageing [9]. Zn supplementation "in vitro" increased Poly(ADP-ribosyl)ation in T-cell clones (TCC) and similar effects were obtained after "in vivo" supplementation in elderly people. The influence of Zn on PARP-1 activity could impact on cellular capacity to repair DNA strand breaks. **María Moreno-Villanueva (University of Konstanz, Germany) **investigated the effect of ex-vivo Zn supplementation on DNA strand break repair, demonstrating that Zn has a highly significant, positive effect on DNA repair if baseline repair is low (perhaps due to Zn deficiency). The influence of Zn on reactive oxygen species (ROS) production in PBMC from healthy elderly subjects, before and after Zn supplementation, was investigated by **Jolanta Jajte (Medical University of Lodz, Poland)**. She found that Zn may play an important role in lowering oxidative stress in cells of elderly subjects [10], although this effect was not uniform and depended on age, gender and Zn status before supplementation. **Efstathios S. Gonos and Ioannis P. Trougakos (NHRF – The National Hellenic Research Foundation, Greece) **showed that also the Clusterin/Apolipoprotein J (ApoJ) gene is responsive to Zn [11]. MT and ApoJ are both involved in cell differentiation, so these results may suggest that the Zn released by MT could affect cell differentiation by affecting ApoJ expression. A positive correlation between ApoJ and Zn was also shown in the plasma of healthy old subjects and an increasing trend was observed post-supplementation. The origin of the differences in the modulation of ApoJ following supplementation are still being evaluated. The fact that MT can be involved in the regulation of ApoJ as well as other Zn-dependent stress-related proteins make it very relevant to understand the changes occurring in the function of these proteins with advancing age. **Marco Malavolta (INRCA-Italian National Research Centres on Aging, Italy) **illustrated the age related changes of these proteins and the interplay between MT protein and mRNA expression as well as their respective modulation by Zn ions and pro-inflammatory cytokines [12]. MT were found to be associated with ageing, cognitive impairment and perceived stress only when there was reduced NO-induced release of Zn, in other words, in the presence of oxidized and perhaps dysfunctional MT. Therefore, the increased MT observed following Zn supplementation as well as in the presence of good Zn status, seems a positive sign generally associated with optimal NO-induced release of Zn and fully functional MT. The hypothesis of dysfunctional MT in ageing was in part confirmed by the results on glutathionylated MT found by **Marco Colasanti (University of Rome 3, Italy) **[13]. The involvement of these proteins in age-related disease as well as their contribution to longevity, was also confirmed by association studies on MT polymorphisms [14], longevity and cardiovascular disease carried out by **Robertina Giacconi and Elisa Muti (INRCA-Italian National Research Centres on Aging, Italy)**. Other targets of the antioxidant Zn response are likely to be methionine sulfoxide reductase (Msr) and the proteasome system. **Bertrand Friguet and Isabelle Petropoulos (Université Paris 7 Denis-Diderot, France) **showed that ageing is associated with a decrease of proteasome chymotrypsin-like peptidase and Msr activities, implicating an impaired response to accumulation of oxidative damage with ageing [15]. In this regard, very relevant results were obtained by Zn supplementation in elderly, as shown by **Filipe Cabreiro (Université Paris 7 Denis-Diderot, France) **in that Zn supplementation improved both proteasome chymotrypsin-like peptidase and Msr activities in the elderly, thus restoring at least in part the intracellular defences against oxidative damage in PBMC [16]. This phenomenon is also evident for chaperone inducibility which, in turn, is markedly reduced in PBMC from old donors [17]. **Csaba Sõti and Ákos Putics (Semmelweis University, Hungary) **demonstrated that Zn supplementation in elderly subjects induced a marked, significant increase in Hsp72 inducibility upon heat stress. Furthermore, Hsp72 induction displayed a strong correlation with Zn bioavailability, suggesting that Zn is a co-inducer of the stress response and a possibly important modulator of the adaptability of the immune system in the elderly. The impact of Zn supplementation on plasma antioxidant enzymes of elderly subjects [18] was studied by **Patrizia Mecocci (University of Perugia, Italy)**. An increased activity of plasma superoxide dismutase and erythrocyte superoxide dismutase, and decreased activity of plasma Cat and GPx were found after supplementation. The reason for such "compensatory" antioxidant enzyme levels is an intriguing clue, perhaps dependent on several factors including genetic background, that will be the object of further investigations. Finally, **Wolfgang Maret (University of Texas Medical Branch, TX, USA) **reviewed the redox biology of Zn and MT, focusing on the control of oxidative signalling by the latter [19]. These signals, estimated in picomolar amounts of Zn, appear to be potent effectors. Amplitudes of Zn signals are determined by the cellular Zn buffering capacity, which is in turn regulated by MT. Therefore, the possible age-associated loss of the ability to make Zn available for insertion into newly synthesized Zn metalloenzymes could be related to the age-related changes in MT and oxidative stress.

## Zn and genomic stability

Some Zn-dependent enzymes involved in the stress response, such as Poly(ADP-ribose) polymerase (PARP-1), are concerned with the maintenance of telomere function and genomic stability. A fast and reliable high-throughput quantitative method to measure telomere length has been developed by **Maria Blasco and Andres Canela (CNIO – Centro Nacional de Investigaciones Oncológicas, Spain)**, by adapting standard Q-FISH to a 96-well format, coupled with automated microscopy [20]. Applying this method, a significant increment of mean telomere length after Zn supplementation was observed in German donors, whereas Italians displayed no changes. These results confirm that inherited and environmental factors may contribute to affect the response to Zn supplementation. In this respect, specific experiments have been performed by **Dawn Mazzatti and Jonathan Powell (Unilever Research Colworth, UK) **by applying nutrigenomic and nutrigenetic approaches to explore the effects of Zn on gene expression profiles [21] of young and elderly people and to correlate the response in PBMC from elderly subjects to IL-6-174 and MT +647 polymorphisms. At least 13 genes were found to be commonly regulated by Zn in elderly and young donors (independently of age), whereas most of the other genes were differentially regulated. A very relevant point was the differential regulation of primarily metabolic pathways in the young compared to a mainly inflammatory response in the elderly. However, a subset of the elderly population with MT1A +647 and IL-6-174 polymorphisms (C+C- carriers) responded very favourably to Zn treatment by reduction in inflammatory cytokine production and altered regulation of energy metabolism. Because these individuals are at high risk for age-related diseases, the anti-inflammatory effects of Zn may represent an important intervention to restore immune function and reduce risk of morbidity and mortality. **Jolanta Jajte and Janusz Blasiak (Medical University of Lodz, Poland) **studied the impact of Zn supplementation on DNA damage and DNA repair in Zn-supplemented elderly subjects using single cell gel electrophoresis assays (Comet assay) [22]. The overall results suggested that Zn supplementation may favour the response of the cells to DNA damage, especially in women above 70 years (an age class with a particularly high risk of Zn deficiency) and that DNA repair processes can be also stimulated by Zn.

## Zn and signal triggering

It is well known that Zn is involved in the mechanisms of cell cycling, proliferation, cell death and signal transduction, but the modulation of these processes following "in vivo" Zn supplementation in elderly subjects is still an open question. **Georges Herbein, Tamas Fulop and Audrey Varin (Université de Franche – Comté, France) **investigated IL-6 and IL-2 signalling [23], focusing on the activation of the STAT pathway in T cells of elderly healthy subjects supplemented with Zn. They showed that when subjects with marginal Zn deficiency were treated with physiological doses of Zn, no changes in Jak/STAT signalling under IL-2 and IL-6 stimulation occur, suggesting that Zn deficiency was not the underlying cause of the signalling defect and correlating well with previous results on in vitro Zn supplementation. The presence of large differences due to a different basal Zn status, country of origin, gender and genetic background, could partially explain the lack of a clear homogeneous response. Conversely, Zn supplementation seemed to have a quite homogeneous effect regarding the modulation of apoptotic mechanisms. **Daniela Monti and Rita Ostan (University of Florence, Italy) **studied the effect of Zn supplementation on mitochondrial membrane depolarization during spontaneous and deoxyribose-induced apoptosis [24]. Their results indicated a clear protective effect of Zn both in spontaneous and induced apoptosis. However, when the possible protective effect of Zn was evaluated in relation with polymorphism (codon 72) of the Zn-dependent factor p53, no difference was observed between Pro-and Pro+ carriers, suggesting that the possible protective effect of Zn on apoptosis is independent of the p53 polymorphism. No effect was observed in cell cycling before and after Zn supplementation except for a slight reduction of G2/M phase after Zn supplementation. Further experiments and clinical trials are necessary to clarify the effect of Zn supplementation in vivo because these encouraging preliminary suggest that this approach could be useful in studying all those diseases with a pathogenesis related to oxidative stress. In particular the involvement of Zn transporters remains scarcely studied. Nevertheless, it is known that the involvement of Zn in the mechanisms of apoptosis and cell cycling is not only due to its release from MT but also from the regulation of its influx/efflux via Zn transporters. **Israel Sekler (Ben-Gurion University of the Negev, Israel) **described the molecular mechanisms of Zn homeostasis, with a particular focus on the molecular identity, function and regulation of Zn transporters [25]. He presented a new hypothesis and results on the regulation of vesicular Zn transporters and Na^+^/Zn^++ ^membrane exchange mechanisms which will surely be the object of future research in this field.

## Zn and immune mediators

Long term clonal cultures represent good models for studying the behaviour of T cells under chronic antigenic stress and facilitate the in vitro testing of interventions in a longitudinal ageing system. This reflects the situation in elderly individuals in which T cells are required to maintain immunity to persistent pathogens, particularly herpes viruses (and especially CMV and EBV), throughout life [26]. These virus-specific T cell clones are sequentially lost during ageing. Longitudinal studies of free-living very elderly people have suggested that such clonal attrition predicts mortality. **Jürgen Kempf and Graham Pawelec (University of Tubingen, Germany) **showed an increased ability of T cell clones cultured long-term in certain concentrations of Zn to synthesise chaperone proteins, following sublethal heat shock. This protective effect of Zn was even more marked when the cells were challenged with a second, higher, otherwise lethal, heat shock. Thus, if these phenomena also occur in vivo, Zn supplementation may facilitate protective hormetic events in chronically stressed T cells and help to prevent the clonal attrition characteristic of the terminal stage of life.

The role of the thymus is vital for orchestration of T cell development and maturation. **Richard Aspinall and Wayne Mitchell (Imperial College London, UK) **evaluated the role played by Zn in maintaining thymic output in healthy supplemented elderly individuals [27]. Analysing subjects from different countries, no significant difference was observed in levels of detectable T cell receptor excision circles (TREC) after Zn supplementation with respect to basal levels. This was found to be the case for both males and females. However, an increasing trend for individuals with particularly low TREC measurements was observed in some countries i.e. males from Italy, Poland and Greece, suggesting that factors affecting individual differences could play a major role. The influence of genetic factors on the immune system was investigated by **Erminia Mariani (Istituti Ortopedici Rizzoli, Italy) **through the assessment of NK activity and the levels of a pool of pro-inflammatory cytokines and chemokines in elderly subjects [28] before and after Zn supplementation. She reported a general increase of NK cell cytotoxicity after Zn supplementation independent of the country of origin but with differences related to IL-6-174 and MT1A +647 polymorphisms. Regarding the inflammatory profile, it was found to be generally decreased in Italian and German supplemented elderly, whereas it was increased in Greek and Polish subjects. Other than the country of origin, many differences were found when considering individual chemokine and cytokine levels in relation to genetic background, basal Zn status and more general dietary habits. These results confirm that Zn is a key trace element for a prompt immune response to external noxae. On the other hand, **Lothar Rink (RWTH Aachen University, Germany) **reported that Zn inhibited basal pro-inflammatory cytokine production but stimulated production after antigen stimulation [29]. He also reported a series of beneficial effects of Zn supplementation observed in the supplemented elderly including reconstituted TH1-cytokine release, decreased spontaneous cytokine production and decreased numbers of activated T cells. All these results were most likely attributed to the increase of intracellular labile Zn in the PBMC of elderly subjects following the supplementation period. Intracellular labile Zn could represent an important marker with possible use in clinical diagnosis of Zn deficiency. The involvement of Zn transporters in modulating intracellular labile zinc during ageing was also discussed. Finally, a comprehensive review of the essentiality of Zn for humans was provided by **Ananda Prasad (Wayne State University School of Medicine, USA)**. He described how Zn deficiency affects immune function and illustrated that what can be called a "Zn deficiency syndrome", affects about two billion people [30]. Thus, correction of Zn deficiency is likely to have a great impact on the health of a large population in the developing world and this could be applicable to the elderly population.

## Zn, nutrition and health

Maternal/fetal nutrition is an important factor in the origin of certain common diseases that affect adults and cause morbidity among the elderly. Contributions of specific micronutrient deficiencies to the phenomenon can be inferred. Because Zn deficiency affects at least 1 in 5 people worldwide, and Zn is essential for many functions [31], not the least of which is the structure/function of thousands of proteins, evidence of the critical importance of Zn for human pregnancy, and effects of Zn deficiency during early development in rats on brain maturation and subsequent function in later life was discussed by **Harold Sandstead (University of Texas Medical Branch, USA). **The essentiality of Zn for nucleic acid and protein synthesis, and certain epigenetic processes, might explain these experimental findings. Zinc nutriture's specific effects of on epigenetic processes are incompletely understood. Here is the problem of how and how much ageing affects Zn homeostasis and requirements. **Sue J Fairweather-Tait (Institute of Food Research, Norwick, UK) **reviewed this aspect showing that the responses to Zn loading measured as changes in plasma Zn, urinary Zn excretion and liver Zn increase with age while the fraction of Zn taken up by red blood cells decreased with age. Additionally she illustrated how Zn absorption is affected by the intake of other nutrients or trace elements [32]. Therefore, a complete evaluation of dietary habits could help to understand the propensity towards Zn deficiency. The Mediterranean diet has been suggested as a strong modulator of the health status and a determinant of longevity. The assessment of adherence to the Mediterranean diet in European old populations and the investigation of its impact on inflammatory and Zn status was addressed by **George Dedoussis (Harokopio University, Greece)**. He showed that in Greece, where the greatest incidence of obesity among elderly was found [33], as well as in Poland, there is a lower adherence to the Mediterranean diet sometimes associated with a lower Zn intake than in the rest of Europe. This issue was further reported in detail for the Italian population by **Cinzia Giuli and Roberta Papa (INRCA-Italian National Research Centres on Aging, Italy)**. The relationship between "Zn status" and psycho-social conditions, especially in countries with the highest propensity towards age-related Zn deficiency [34], was illustrated by **Fiorella Marcellini (INRCA-Italian National Research Centres on Aging, Italy)**. She observed also a slight but beneficial effect of Zn supplementation on cognitive functions of elderly subjects supplemented with Zn. Taking into account that most of these elderly subjects carried specific alleles for IL-6-174 and MT1A +647 polymorphisms, these results further confirm that the response to Zn intake/supplementation can be affected by the genetic background of each individual.

## Conclusion

Zn research in the context of ageing is still in a relatively early stage. Several improvements in sensitivity and specificity of methods to assess Zn status have been developed, but severe limitations in reproducibility still exist. Nevertheless, new knowledge on the function of metallothioneins and Zn transporters in ageing has been acquired. Recent results obtained with Zn supplementation in elderly subjects are encouraging. Many factors were found to affect the individual response to Zn, such as general dietary habits, genotype, gender, drug usage and frailty. This makes it very difficult to draw a definitive conclusion regarding the possible benefits of Zn supplementation during ageing. However, some progress in understanding how Zn can modulate oxidative stress responses and host defence to infection has been achieved and will hopefully be the subject of further scientific enquiry. It is also clear that in order to clarify many aspects related to the health benefits of Zn, a longitudinal approach will be helpful using cohorts of subjects whose past dietary habits and clinical data can be easily accessed.
